# Diphtheria*Read before the North Texas Medical Association December 7-8, 1915.

**Published:** 1916-02

**Authors:** M. L. Wilbanks

**Affiliations:** Greenville, Texas


### Diphtheria.*

BY M. L. WILBANKS, M. D., GREENVILLE, TEXAS.

In discussing this very common but very distressing disease, let us see it from the standpoint of the ancients, and in this discussion the definition that was given the disease in ancient times will suffice to explain that it is now just as it was then.

Diphtheria is a contagious disease, directly transmissible from person to person, also indirectly from a third person and infected fomites. There is not a disease more amenable to treatment that the physician has to deal with, and the main part of this paper will be to impress on the minds of the doctors the necessity of giving their careful attention to every sore throat that comes within their knowledge.

From the names as gathered from ancient history for a condition that is in all probability what is now known as diphtheria, we readily see that the people were greatly frightened at the sight of any case so infected. In Egpyt it was known as "morbus Aegyptiacus" and "syriacus," and Spanish people called it "Garotillo," after the cudgil of the executioner, and Swedish people called it "Strypsjuka" (strangling disease). It was not until the first century A. D. that an accurate clinical description was given, and found in the writings of Aretaeus, a Greek physician; in England, by Fathergill, in the latter part of the eighteenth century, and by Ward in America about the same time, but it was

*Read before the North Texas Medical Association December 7-8, 1915.

Bretanneau, in 1826, that gave it the name of diphtheria. The organism now known as Klebs-Loefler bacillus were isolated and described by Loefler after repeated attempts had been made along the same line by Klebs. The toxin was next determined by the works of Roux, Yersin and others, and finally the antitoxine was discovered by Behring.

There are a number of recognized modes of infection: (a) From person to person. (b) Fomites may harbor the bacillus for many months, some claim. (c) Persons suffering from atypical forms of diphtheria, nasal catarrh, membranous rhinitis, and some cases are on record of the membranous colon which have thrown off membranes that contained the bacillus. (d) From the throats of healthy persons, diphtheria carriers, if you please, persons who harbor pathogenic bacteria in their bodies, without suffering themselves. This may be because they have just recently recovered from the disease or that they have acquired the organism by association with one who has the disease. Diphtheria was the first disease in which carriers were recognized, and it remains the particular disease in which the carriers are of the most importance in its propagation. The most modern teaching is that this disease is only transmitted by personal contact, though there are some who do not think so. Fomites and room infections are things of the past, as taught by some, as well as that of the air, and the latter is only a medium so far as the zone of droplet infection extends around the person supplying the virus. It is therefore evident that the carriers are of the utmost importance from the standpoint of prevention.

It is claimed by some that from 1 to 7 per cent of urban communities are carriers of this disease. In Chicago recently when 20,000 school children were examined 600 were found to be contaminated with diphtheria bacteria, as was found from smears from discharges from their throats. This will explain why we have outbreaks without any apparent case from which develop the disease. There is not a physician here who has not seen cases develop, sporadically, to which no trace of infection can be made, unless an examination of the throats of those to whom this particular case has been exposed.

The question, then, of prevention is hindered in the rural places because of this one cause, that of carriers.

The clinical symptoms of this disease are so varied and uncertain that to say this child, or that, has diphtheria or has not, in differentiating from the other ulcerative throats, is only to be made by the demonstration of the bacillus, and there is not a man here who has not seen a time when it was dangerous to wait, though there were no membranes in the throat. The marginal cases—those that simulate other throat affections—are the sources of the epidemics in the communities; and it is here that the many physicians allow the spread of the disease by failing to recognize it. To illustrate: Mrs. B. brought her child to my office some two months ago with a suspicious patch on both tonsils, a temperature of 101°, pulse 100. The father refused the administration of the antitoxine. Called in another physician, who did not corroborate my diagnosis, and so let the child go untreated with the antitoxine, which I had suggested and which I had said without which I could not continue in the case and take the responsibility. The final end result of this case was that the other child in the family was stricken after the former one had gone through a long siege of otorrhea and other nasal complications for weeks and weeks, to be followed by the development of the diphtheria in this child and the mother as well. The second child died after every effort to save him had been made, and the mother was stricken but recovered.

Some have suggested that there are some certain signs by which this disease can be recognized, such as if the temperature is found higher than 101° in any child suffering from a sore throat can safely be called tonsilitis, and any case below that may be called diphtheria. I admit that there is a strikingly high temperature in cases of tonsilitis, but this is not to be taken as infallible. The smell is a very characteristic symptom. No other odor like it, but not infallible. The membrane is a most certain sign; however, not every case that has no membrane is negative; for we have the laryngeal type without any membrane visible. When the membrane appears on the tonsils, uvula or post nares, one can usually be safe in pronouncing the case diphtheria. Post-tonsilectomies have been mistaken for the disease, and it would be well to know if the tonsils have been removed a few days previous to this examination. The manner of breathing in a laryngeal diphtheria is very characteristic and is a retroaction of the epigastrium. In this form we may mistake it for pneumonia and our patient may die of toxemia without it having been recognized until some exposed member of the family falls a victim. In all cases of nasal discharge there should be a suspicion of diphtheria.

Having been confronted with a case of diphtheria, either absolute or suspicioned, the use of antitoxine is the remedy per se, and with this we all agree, though it has not been long since we had objectors to its use. One who now objects and refuses to administer antitoxine in a case of diphtheria is a murderer, and should be dealt with as a criminal.

Granting that all are in favor of the administration of the antitoxine, yet there seems to be no means of alleviating human misery that enjoys so wide a range of ideas as to its administration, not only in the mode but in the amount that it takes to relieve, as does diphtheria.

My personal experience is that the earlier and the larger the dose the better. However, when the dose is given that is larger than is required to overcome the toxine in the blood of the particular individual, we have lost that amount of excess antitoxine. No therapeutic dose of less than 5000 units to any child should be risked, in my judgment, and if the child is over two years old 10,000 is better. When the larger dose is given there will, in all probability, not be a necessity for the second dose, if seen and given early. A safe rule is 500 units to the kilogram of weight of the child. As an immunizing dose, 50 to 100 units to the kilogram will suffice.

Schick recommends that the antitoxine be given early and intramuscularly, claiming that it is absorbed more rapidly in this manner. It is claimed by other good men that the most prompt results are gotten from the intravenous injection.

I think that the subsequent injections are superfluous, let it be either intracellular, intramuscular or intravenous, after sufficiently large initial dose is given in the mild case as above stated, 5000 to 10,000 units, and in the severe case as much as 30,000. If the diphtheria patient dies, it is because the toxin elaborated by the organisms in his body has had time to act before the antitoxine was injected, and cannot, by any means as yet known to us, be afterward inactivated. The only circumstance in which the second dose is of value is where the initial dose has been less than as recommended here; then one may give a single dose of 500 units per kilogram. I have given repeated doses myself and have had reactions from some of the larger repeated doses, as nettle rash in some cases, where much as 30,000 units have been given in repeated doses.

The same notations have been made in the intravenous injection of antitoxine as has been made with quinine in malaria, that neither give the skin manifestations that former doses of quinine, given by the mouth, gave and that of antitoxine intracellular or muscular.

Having given our patients the antitoxine and other attention that is necessary for their welfare, we will have some chronic complications that will act as carriers and have to be taken care of in order to prevent the further contamination of our schools and homes.

We will have the different catarrhal affections follow: otitis media, nasal passages flowing in pus, the expulsion of which will enhance the chances of the otherwise immune, exposed and subsequently an outbreak that was thought to be under control. There seems to be a prevalency of opinion that autogenous vaccines eradicate this fountain of infection very rapidly, not in every case but in a large per cent.

Dr. Weil reports some autogenous vaccine cases beginning with 160 millions, increasing in five doses up to fourteen hundred million. He made another observation that will be very helpful in this vaccine, and that is that though the vaccine contain much toxin, yet no reactions of importance were noticed.

Diphtheria, it is important to remember, cannot any longer be regarded as a purely local infection. The bacillus may be found in the organs of diphtheria patients, and have been found even in the urine.

Dr. Cargin reports a case that had had faucial diphtheria and had recovered with the usual dose of antitoxine. Recovery was interrupted in about a week with abdominal pains, whereupon soap and water were used and washed away several pieces of membrane, a portion of which was apparently part of the small gut. Upon the use of antitoxine the symptoms all disappeared.

There is one plan of treatment that I will mention, but to condemn it with all the force that is at my command because: First, it is unscientific, inhuman, unreliable and satisfies the user that something of importance is being done; all the while the patient is dying with toxemia, which is enhanced by suffocation. I have been guilty and have an intubation set myself. Why should the intubation of a child, suffering with diphtheria, be discarded? In all cases of suffocation, not in this disease, but in every case, there should be provided a safe and permanent chance for this patient to obtain that oxygen that is so necessary to life and the expulsion of the toxines. The danger of suffocation—and it is great, is increased with the introduction of an intubation tube; it is liable to be coughed out and the patient will be where the was at its introduction, except weaker.

To forcefully bring this to your minds, I will recite an incident that came under my observation. A child was brought by a physician seven miles from the country, over rough roads on a cold afternoon to town to have an intubation. This operation having been performed by one who is dexterous in the art, the child and family returned to their home. In a very few hours the child coughed the tube out, and the same run had to be made again. What shall we do for these suffocating children? We will have them if we have any diphtheria. A country physician did a tracheotomy with his pocket case and used a fountain syringe nozzle as a tube, and the patient recovered. The simple operation of a tracheotomy is by far the safer and more scientific to perform. Mr. A. Baginsky, Berlin, says this about intubation: 'Intubation, it is true, is not an easy operation, but must be practiced. In the hands of an unskilled operator, the intubator, armed with the tube, is a dangerous instrument which may cause death by producing injuries. If the tube produces the injury the child most often dies of broncho-pneumonia. This must be remembered, and no one should attempt intubation without having practiced on the cadaver."

Of course there are objections to tracheotomy, for there are instances where the membrane forms on the wound and is slow to heal, though some wounds be weeks in healing, yet, that is preferable to death by strangulation.

To recapitulate: The disease is transmissible principally, if not altogether, by personal contact. The history of its ancient heritage proves that the disease is transmitted by carrier beyond doubt, as shown by the vast number of children who are perfectly well but who are possessors of the bacteria in either their throats, their ears or their bowels.

From 1 to 7 per cent of the school children in urban communities are carriers.

Any child suffering with a sore throat, with a temperature running lower than 101.2° should be held very suspicious, though higher temperatures are seen, but not as a rule. All cases with tonsilitis should be treated suspiciously if there is any diphtheria in the community.

The size of the dose of antitoxin should not be less than 5000 units for children under 2 years, and as they are older relatively larger. A safe rule is that 500 units per kilogram of weight of the child. If given early, and the size sufficient, second injections are superfluous. In many cases there are sequelae of different affections of the nasal passages which might be expected but prevented. For these cases, autogenous vaccines are recommended.

Diphtheria is no longer considered a purely local condition. As intubation is unscientific, inhuman, unreliable and only satisfies the physician using it, and as there is great danger of suffocation in its attempted use and, if it is successfully used, there is danger of the tube being coughed out, tracheotomy is suggested as the more rational treatment when necessary. By using larger doses of antitoxin, neither intubation or tracheotomy will have such places as they have had under the old régime. Tracheotomy is not without its objectionable features, however.

(For much of the data herein contained I am indebted to Mrs. F. E. Daniel's Medical Package Library.)

